# Recent Developments and Challenges of Edge Termination Techniques for Vertical Diamond Schottky Barrier Diodes

**DOI:** 10.3390/s25226974

**Published:** 2025-11-14

**Authors:** Genzhuang Li, Wang Lin, Shishuai Liu, Yeldos Aileplanm, Aochen Du, Liuan Li

**Affiliations:** 1 Xinjiang Laboratory of Phase Transitions and Microstructures in Condensed Matter Physics, College of Physical Science and Technology, Yili Normal University, Yining 835000, China; 2 Yili Engineering Research Center of Green Silicon-Based Materials, College of Physical Science and Technology, Yili Normal University, Yining 835000, China; 3 Tianjin San’an Optoelectronics Co., Ltd., Tianjin 300384, China; 4 Tianjin Key Laboratory of Semiconductor Light Emitting Diode Chip Enterprises, Tianjin 300384, China; 5 State Key Laboratory of High Pressure and Superhard Materials, College of Physics, Jilin University, Changchun 130012, China

**Keywords:** diamond, Schottky barrier diodes, edge termination structures, power electronic devices

## Abstract

Thanks to its excellent material properties, diamond-based power electronic devices have garnered widespread attention. The realization of large-sized (over 2 inches) and high-quality single-crystal diamond wafers has significantly accelerated the industrialization of diamond semiconductor materials and devices. Over years of development, diamond Schottky barrier diodes (SBDs) have evolved into three primary device structures: lateral conduction type, quasi-vertical conduction type, and vertical conduction type. However, the performance of these devices has yet to fully unlock the potential of diamond materials. Efficient edge termination structures need to be designed to synergistically optimize the forward turn-on voltage, on-resistance, and off-state breakdown voltage. This paper reviews the research progress on various existing edge termination structures of diamond SBDs, analyzes the advantages of each structure, and discusses the key challenges faced in the device fabrication processes.

## 1. Introduction

Power devices, which rapidly convert electrical energy from one form to another, represent a major breakthrough innovation in power systems [1]. Over the past few decades, silicon (Si)-based power devices have played a dominant role in electrical energy utilization. In the next 20 years, energy consumption from electricity is expected to account for about 60% of total energy consumption, and this proportion is expected to increase. However, traditional Si-based devices are gradually approaching their theoretical material limits [2]. Therefore, developing power devices based on new materials and structures to continuously improve their power density and operating frequency is fundamental for efficient utilization of electrical energy. The wide bandgap semiconductors, such as GaN, SiC, Ga_2_O_3_, and diamond, have become one of the most promising materials for next-generation power electronics and optoelectronics [3]. Compared to traditional Si material, the diamond exhibits numerous advantages, such as high carrier mobility and thermal conductivity, low thermal expansion coefficient, and high critical electric field, making it suitable for high-voltage applications such as high-power generators and inverters in trains, ships, renewable energy systems, and power grid systems. The development of diamond power devices meets the major social demand of carbon neutrality. Due to the ease of obtaining large-sized single-crystal substrates, research on GaN, SiC, and Ga_2_O_3_ power devices has made rapid progress. The power diodes employing those materials have realized the kV-class breakdown voltage (*V_BD_*) with on-resistance (*R_on_*) in the mΩcm^−2^ level [4,5]. In recent years, large-sized (over 2 inches) and high-quality single-crystal diamonds (SCDs) have been successfully produced through mosaic growth or heteroepitaxy techniques [6,7,8,9], greatly promoting the industrialization of diamond semiconductor materials and devices.

**Figure 1:**
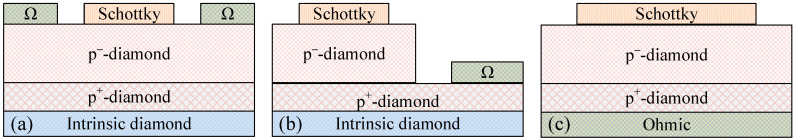
Schematic diagram of the basic device structures of diamond SBDs, (**a**) planar type, (**b**) quasi-vertical type, (**c**) vertical type, respectively.

Diamond power diodes primarily include Schottky barrier diodes (SBDs) and PN junction diodes (PNDs) [10]. SBDs have broad application prospects in the kV-class area due to their advantages, such as low turn-on voltage (*V_on_*), low recovery current, and high switching frequency. Until now, p-type conductive diamond obtained through boron-doping is relatively mature because of its small atomic radius and high solubility. The electrical parameters (widely adjustable doping concentration and high mobility) also generally meet the device requirements [11]. Currently, the fabrication of diamond SBDs mainly employs boron-doped epitaxial layers. Depending on their structures, they can be mainly divided into three types: lateral conduction type, quasi-vertical conduction type, and vertical conduction type (Figure 1). The lateral conduction structure (Figure 1a) typically uses high-pressure high-temperature or chemical vapor deposition-synthesized intrinsic diamond as the substrate. Since the substrate is non-conductive, both ohmic contacts and Schottky contacts are fabricated on the diamond drift layer. This structure has a simple preparation process, but the edge of the Schottky electrode often experiences electric field crowding effects, which degrade the device’s off-state *V_BD_*. Usually, increasing the electrode spacing is required to enhance the device’s *V_BD_*, but this also significantly increases the forward *R_on_*. The vertical conduction type device is a solution to address the aforementioned challenges [12]. It can increase the drift layer thickness to enhance the *V_BD_* while maintaining the device size, thereby avoiding premature breakdown caused by the electric field crowding effect in lateral devices. Additionally, the electric field distribution of vertical devices is relatively more uniform, and the current distribution is more spread out, resulting in higher power density and superior heat dissipation characteristics. However, growing a boron-doped p-type diamond epitaxial layer on intrinsic diamond material is less challenging than growing a p-type diamond substrate, and a quasi-vertical structure is often adopted as a compromise (Figure 1b). Compared to the lateral conduction structure, the diamond epitaxial layer needs to be etched (mostly dry etching) to the p^+^ layer to form a mesa structure. In this structure, current flows from the Schottky electrode through the p^−^ layer, p^+^ layer, and then to the ohmic contact electrode, with a current crowding effect at the edges of the Schottky and ohmic electrodes. Generally, the potential of materials in semiconductor power device applications is usually evaluated using the Baliga figure of merit (BFOM) value, which can be described as follows:

(1) $$BFOM=\frac{4V_{BD}^{2}}{{\varepsilon_{s}\mu }_{h}R_{on}}$$

where $\varepsilon_{s}$ is the dielectric constant, and $\mu_{h}$ is the mobility of a hole.

To realize a relatively higher *V_BD_*, it requires a thicker drift layer and a lower doping concentration. However, this may cause an increase in *R_on_*. Therefore, it is necessary to balance those parameters to achieve the highest BFOM values. With the breakthrough in the preparation technology of large-sized, low-resistance p-type diamond substrates, Schottky diodes with a vertical conduction structure (Figure 1c) are more conducive to synergistically optimizing the *V_on_*, *R_on_*, and *V_BD_*, thereby achieving a higher BFOM value.

## 2. Improvement in Properties for Diamond SBD

**Figure 2:**
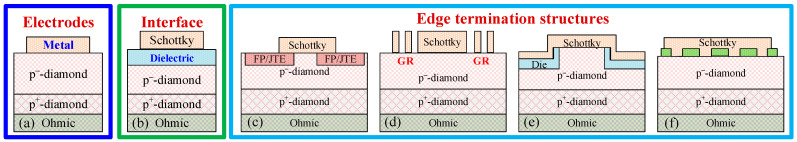
Typical strategies used for vertical diamond SBDs to enhance the electrical performances. (**a**) electrode material selection, (**b**) interface engineering, and (**c**–**f**) edge termination structures.

Currently, the performance of SBDs is primarily regulated through three technical approaches: Schottky electrode material adjustment, interface engineering, and edge termination structures, as illustrated in Figure 2.

### 2.1. The Effects of Schottky Metal Material

In 1997, A. Vescan et al. fabricated the first diamond SBD operating at 1000 °C, consisting of a boron-doped p-type (100) diamond and a Si Schottky electrode [13]. The fabricated device achieved a rectification ratio of 10 but exhibited a low breakdown voltage. In 2014, A. Traoré et al. fabricated a quasi-vertical diamond SBD with Zr as the Schottky electrode [14]. At a Schottky contact area of 7.85 × 10^−5^ cm^−2^, a current density of 10^3^ A/cm^2^ (@6 V) and a breakdown electric field value greater than 7.7 MV/cm were achieved. This is also the device closest to the theoretical critical electric field of diamond to date. In 2015, S. Tarelkin fabricated a vertical diamond SBD with Pt as the Schottky electrode [15]. The forward current exceeded 10 A at temperatures ranging from 25 to 200 °C. Simultaneously, the forward voltage at 10 A decreased from 3.5 V (at 25 °C) to less than 1 V (at 200 °C). In 2015, K. Ueda et al. fabricated a lateral diamond SBD with Cu as the Schottky electrode [16]. The *R_on_* and *V_BD_* of the device at 400 °C were 83.4 mΩ cm^2^ and 713 V, respectively. In 2022, N. Saha et al. fabricated a diamond SBD device with Ti as the Schottky electrode [17], exhibiting a rectification ratio of 3.6 × 10^5^ and a high *V_BD_* of 1650 V. However, the currently obtained devices exhibit significant reverse leakage current, resulting in a device breakdown electric field far below the material limit. Typically, metals with relatively low barrier heights are selected as Schottky electrodes to reduce the *V_on_*, which simultaneously leads to relatively high reverse leakage current. Altering the work function of metal electrodes to increase the barrier height is the primary means of suppressing leakage current. However, numerous experimental results have demonstrated that the Schottky barrier height of oxygen-terminated boron-doped diamond has no significant dependence on the surface oxygen treatment methods and the types of metal materials selected (Fermi level pinning) [18]. The Fermi level pinning phenomenon occurs due to surface reconstruction or incomplete oxidation when the hydrogen-terminated diamond surface transitions into oxygen termination. The coulomb interaction of a large number of dangling bonds, defect states, etc., fixes the Fermi level at a position approximately 1/3 of the bandgap width above the valence band maximum (approximately 1.5 eV for diamond).

### 2.2. Interface Engineering by the Dielectric Interlayer

**Figure 3:**
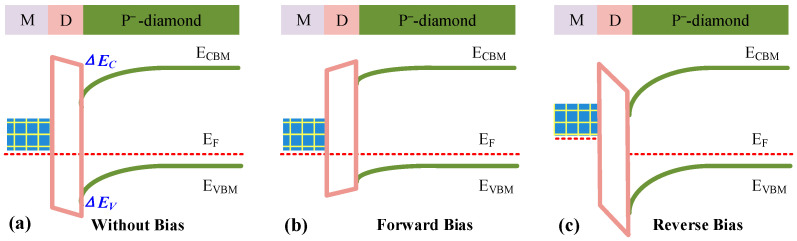
Schematic diagrams of the energy band of the metal/dielectric layer/diamond SBD under (**a**) zero bias voltage, (**b**) forward bias voltage, and (**c**) reverse bias voltage, respectively.

Recently, researchers have reported adjusting the barrier height by inserting a thin layer of dielectric between metals and semiconductors (Figure 3a) [19]. On the one hand, oxygen on the diamond surface re-bonds with atoms in the dielectric layer during the deposition, thereby alleviating the Fermi level pinning effect. On the other hand, the band structure between the metal/dielectric/semiconductor can also affect the interface barrier of the Schottky junction. Typically, the introduced dielectric layer needs to form a certain valence band offset (*ΔE_V_*) with the diamond, thereby increasing the effective barrier height of the Schottky interface. Compared with the Schottky contact without dielectric, the positive *ΔE_V_* increases the barrier height during forward conduction, resulting in a slight decrease in the diode’s current density at the same forward conduction voltage (Figure 3b). While under the off-state, holes movement through the metal/semiconductor interface are also blocked by the high potential barrier, which can effectively reduce reverse leakage current (Figure 3c). Therefore, if the absolute value of *ΔE_V_* remains sufficiently small it can maintain good *V_on_*, low *R_on_*, and high *V_BD_*.

In 2022, Zhang et al. fabricated SBDs by inserting a thin SnO_2_ layer to improve the barrier height to approximately 1.84 eV and increase the *V_BD_* from 102 V to 123 V [20]. Furthermore, the density of interface states was also decreased by two orders of magnitude after the addition of SnO_2_. In 2023, Li et al. systematically studied the effect of the rapid thermal annealing process on diamond Schottky diodes with a thin LaB_6_ interlayer [21]. After annealing at 350 °C, the forward current density, rectification ratio, *R_on_*, and *V_on_* of the device were 2 kA/cm^2^, 1.7 × 10^10^, 0.79 mΩ cm, and 2.3 V, respectively. Although the dielectric layer in this structure increased the forward barrier height to some extent and reduced the reverse leakage current, diamond materials lack intrinsic oxides/nitrides. During the heteroepitaxy process, the dielectric layer faces challenges such as poor adhesion and high stress on the one hand. On the other hand, factors such as lattice mismatch can easily lead to a large number of bulk defects and interface defects, which can capture/release carriers under external stress conditions such as electric fields, resulting in degradation of electrical performances.

### 2.3. The Termination Structure for Diamond SBD

The ideal semiconductor material (without defects such as dislocations and impurities) has a critical electric field, which is approximately 10 MV/cm for diamond material. After a series of optimizations of the Schottky electrode and interface materials, the forward characteristics and stability of diamond SBDs have been greatly improved. However, the electric field crowding effect can easily lead to local electric field strength exceeding the critical electric field, resulting in increased leakage current and premature breakdown of the device. It is worth noting that the maximum electric fields of the recent diamond SBDs are still much lower than the critical electric field (commonly below 5 MV/cm), which can be ascribed to the crystalline quality of the diamond is not perfect. The use of advanced edge termination structures to alleviate electric field crowding is a key method of improving SBD performance. PN junctions are commonly used as termination structures in power SBDs such as silicon, silicon carbide, and gallium nitride. PNDs have a wider depletion region than SBD and relatively smaller leakage current during reverse bias, making them potential candidates for high-voltage applications. The conductivity modulation effect of minority carriers during forward conduction of PND can reduce the forward conduction resistance to a certain extent, but its high forward conduction voltage (>5 V) will increase the forward conduction loss of the device. Therefore, using a PN junction as the termination structure can not only maintain the good forward conduction performance of SBD, but also introduce the good reverse blocking performance of PND. However, there is a serious doping asymmetry challenge in diamond semiconductors. On the one hand, p-type doped diamond with high doping concentration and good activation has been obtained, while n-type doped diamond still poses difficulties in forming effective donors. On the other hand, p-type doping usually adopts the large-sized and high-quality (100) crystal planes diamond, while n-type doping is more easily achieved on (111), (113), and other crystal planes with smaller size and lower crystal quality. Therefore, the currently reported termination structures mainly use methods such as field plates (FPs), high-resistance regions, guard rings (GRs), and MESA structures to replace n-type doping. With the development of efficient n-type doping technology or the introduction of other n-type materials, new devices or termination structures based on PN junctions, such as Schottky PN diodes (SPNDs), junction terminal extensions (JTEs), and junction barrier Schottky diodes (JBS), have gradually been developed.

#### 2.3.1. Field Plate Structure with Dielectric

**Figure 4:**
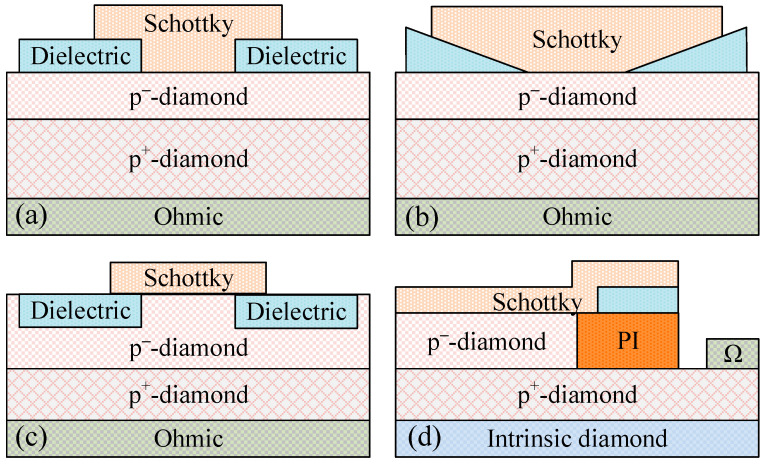
Schematic diagrams of different types of field plate structures. (**a**) The conventional structure, (**b**) ramp FP structure, (**c**) planar mesa field plate structure; (**d**) trench field plate structure.

**Figure 5:**
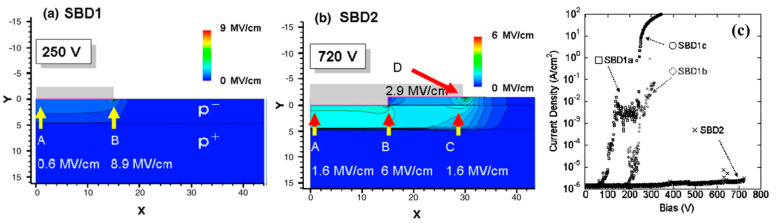
(**a**) and (**b**) are the simulated electric field distributions of SBD without and with a field plate structure, respectively. (**c**) is the corresponding measured current–voltage curves under a reverse bias. Reprinted with permission from ref. [24].

The most common field plate (FP) structure is shown in Figure 4a, in which the dielectric layer is introduced at the Schottky electrode edge with an overlap [22]. When the reverse bias is added to the FP electrode, a depletion region is generated in the p-type drift region located beneath, which is equivalent to the lateral spreading of the original depletion layer of the Schottky junction. Therefore, the electric field at the edge of the Schottky junction is reduced, and the breakdown voltage is improved. In 2008, K. Ikeda et al. simulated the effects of FP by using the materials with low (SiO_2_) or high (Al_2_O_3_) dielectric constant [23]. Both kinds of FPs can modify the electric field distribution and enhance the breakdown voltage effectively. In addition, the Al_2_O_3_ FP with a higher dielectric constant provides a relatively uniform electric field distribution and a lower electric field concentration. Figure 5 shows the simulated electric field distributions of SBD without and with a field plate structure. For the SBD without FP under a reverse bias of 250 V (Figure 5a), the electric fields at the center and the corner of the Schottky contact are approximately 0.6 MV/cm and 8.9 MV/cm, respectively. The crowding at the corner contributes to the premature breakdown in the diamond SBD (Figure 5c). While for the SBD with a FP structure, the maximum electric field was about 6 MV/cm even under a bias of 720 V (Figure 5b) [24]. Recently, a vertical diamond SBD exhibiting a high breakdown field (4.04 MV/cm) and low leakage current at high reverse bias was demonstrated by adopting high dielectric constant SnO_2_ as FP (*ε* = 17) [25].

However, an electric field peak in this structure still occurs at the edge of the Schottky metal–semiconductor interface. One of the solutions is regarded as the ramp FP structure, as shown in Figure 4b. Another solution is regarded as the planar mesa termination structure, as shown in Figure 4c. The diamond mesa region is surrounded by the dielectric buried in the trench structure [26]. The simulation results demonstrate that the lateral depletion region can effectively reduce electric field crowding at the corner of the Schottky contact. The proposed SBD presents an obvious improvement in reverse bias performances while only a minimal effect on forward bias performances. In 2015, Arbess et al. designed a new topology termination by Sentaurus [27]. A trench structure was introduced under the field plate and filled with polyimide (Figure 4d). The relatively smaller permittivity of PI (≈3) than that of diamond (≈5.7) is beneficial to spread the equipotential lines and decrease the maximum electric field at the edge of the field plate.

Although the FP is a simple process and effective in suppressing the electric field crowding, there are still some challenges for this strategy. Firstly, the FP introduces an extra parasitic capacitance in the SBD, which will degrade the high-frequency application. Secondly, owing to the lack of native oxide, there is commonly an interfacial layer with a thickness of 0.5–1.0 nm that exists at the heteroepitaxy interface on diamond. In addition, oxide vacancies are also usually confirmed in the atomic layer-deposited Al_2_O_3_, resulting in the high density of positive charges. Then, high deposition temperature and/or post-deposition annealing are required to improve the interface quality [28]. Thirdly, there is a trade-off between the dielectric constant and bandgap. Generally, the bandgap decreases with the increasing dielectric constant, which means a small band offset (hole barrier) versus diamond and a high tunneling current. Thus, the dielectric for the FP structure of diamond SBD must be carefully chosen.

#### 2.3.2. Guard Ring Structures for Diamond SBDs

**Figure 6:**
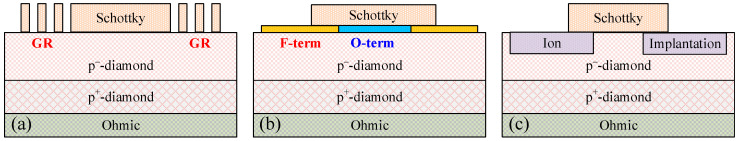
Schematic diagrams of guard ring structures fabricated with (**a**) metal rings, (**b**) hybrid surface terminations, and (**c**) high-resistance ring.

Guard ring (GR) structures, consisting of one or more floating rings around the Schottky electrode (Figure 6), adjust the spatial distribution of the depletion region to disperse the electric field crowding and improve the *V_BD_*. Generally, the PN junction GRs with doping types opposite to the drift layers or Schottky junction rings composed of metal electrodes can be used. Diamonds with efficient n-type doping are difficult to achieve, and current research mainly uses metal to prepare GR structures (Figure 6a). There are two main methods for designing the parameters of GR. The first design is that the ring width and ring spacing are not equal, but the sum of spacing and width is a constant, with the ring width gradually decreasing from the inside out and the corresponding spacing gradually increasing. The second design is that the ring spacing is equal to the ring width, which can reduce the size of the termination region and obtain a better gradient depletion region. In 2018, K. Driche et al. prepared a quasi-vertical SBD with a floating metal GR structure [29], and demonstrated that a multi-metal ring can disperse the electric field crowding and improve the *V_BD_* of the device. In 2019, Wang et al. prepared a diamond SBD with floating GRs [30], and found that the number, spacing, and width of the metal rings themselves all have an impact on the *V_BD_* of the device. The addition of GR does not affect the forward current density and rectification ratio of the device. The GR structure can use the same metal as the Schottky contact, and the process is simple. However, the barrier height between oxygen-terminated diamond and metal is not easy to modulate. But a unique property of diamond is that its affinity/work function can be adjusted by changing the type of surface termination atoms. In 2018, Zhao et al. used F plasma to treat the area around the Schottky electrode on the boron-doped diamond surface to change the oxygen to a fluorine termination (Figure 6b), with a portion of the Schottky electrode covering the fluorine termination region [31]. The Schottky metal forms a barrier height of 2.0 eV with the oxygen termination, which is beneficial for reducing the forward *V_on_*. The barrier height formed with the fluorine termination is 2.39 eV, which is conducive to forming a wider depletion layer width and reducing leakage current. Although the GR technology has a significant effect on improving the *V_BD_*, the parameter design is relatively strict, and it is also very sensitive to interface charges. When the power class is high, it occupies a large surface area of the chip and results in a large reverse leakage current.

The high-resistance region termination structure is achieved by ion implantation with high-energy ion beams to destroy the diamond lattice and generate deep-level defects, ultimately leading to the high-resistance state of the implantation region and regulating the distribution of the electric field (Figure 6c). On the one hand, the applied bias will uniformly drop in the high-resistance region. On the other hand, the prepared Schottky electrode presents an overlap with the high-resistance region, thereby producing a similar effect to the FP to a certain extent. In 2019, Zhao et al. prepared a diamond SBD using a high-resistance region formed by F ion implantation [32]. The *V_on_* and *R_on_* of the device were 1.6 V and 50.2 mΩ cm^2^, respectively. At the same time, the reverse electric field of the device at a *V_BD_* of 117 V was 3.3 MV/cm, which was better than the reference device without a termination structure. In 2019, Yu et al. prepared diamond SBDs using high-resistance regions formed by B ion implantation [33]. The test results show that the high-resistance region does not contribute to the forward current, and a current density of about 4000 A/cm^2^ is obtained when the device is forward biased at 5 V. The reverse *V_BD_* of the device is also increased from 79 V to 125 V. In 2023, Li et al. prepared diamond Schottky diodes by selectively growing low-conductivity nitrogen-doped diamond around the Schottky electrode [34]. The maximum *V_BD_* of the device has been increased from 80 V to 112 V. At the same time, the current density of the device has decreased from 9189 A/cm^2^ to 8742 A/cm^2^ due to the decrease in conduction area. One of the challenges of this solution is the high cost of ion implantation equipment. In addition, the high atomic density of diamond leads to lattice damage in the implantation area, which increases the leakage current at a relatively lower reverse bias. Therefore, the top priority should be to analyze the leakage mechanism by measuring the temperature-dependent current–voltage characteristic curve, and to mitigate lattice damage by combining ion implantation parameters with post-annealing process parameters.

#### 2.3.3. Mesa Termination

**Figure 7:**
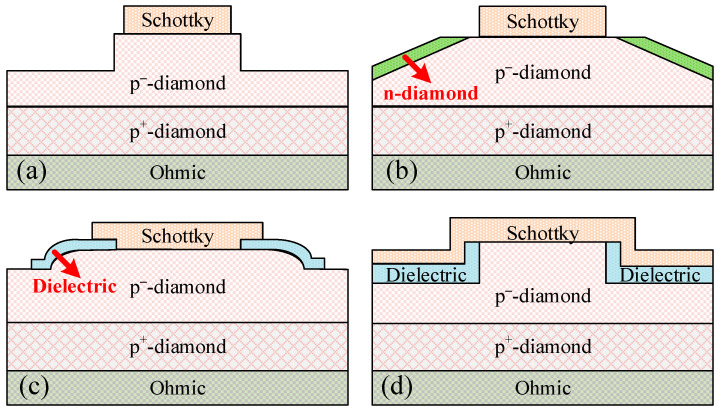
Schematic diagrams of different types of Mesa Termination structures. (**a**) the deep etched MESA, (**b**) beveled MESA with PN junction structure, (**c**) beveled MESA with a certain curvature, and (**d**) trench MOS barrier Schottky (TMBS) structure.

Forming a MESA structure by etching or other methods to change the shape of the electrode edge is a common termination method in PN junction diode devices. It is usually etched into MESA, with a beveled angle, curved surface, and other shapes to improve the surface electric field distribution. The deep etching scheme can homogenize the electric field at the edge of the Schottky electrode (Figure 7a), but both dry etching and chemical etching face certain process challenges for diamond material. Diamonds have high hardness and chemical inertness, and commonly used dry etching can easily cause lattice damage on the surface with a relatively lower etching rate.

The beveled MESA [35] is divided into positive bevel angle (junction area gradually decreasing from heavily doped region to lightly doped region) and negative bevel angle (junction area gradually increasing from heavily doped region to lightly doped region). The surface space charge region of the positive bevel angle termination is relatively wide, and there is no avalanche center caused by high electric field strength. A larger positive bevel angle (between 30° and 80°) can obtain a *V_BD_* close to that of the bulk material. However, it is relatively difficult to process diamond materials with a positive bevel angle. On the other hand, the space charge region width on the surface of the negative bevel angle is greater than that inside the bulk, which can effectively reduce the surface electric field strength. Usually, a decrease in the bevel angle increases the space charge region width and increases the *V_BD_* accordingly. But the smaller the inclination angle, the more surface area occupied by the junction termination of the chip. In 2022, Li et al. simulated a novel diamond SBD with a beveled PN junction termination structure (Figure 7b) [36]. The Schottky contact is also located on the (100) crystal plane of p-type diamond, and the forward *V_on_* of the device remains almost unchanged. However, the depletion region formed by the PN junction will hinder the current conduction range and slightly increase the *R_on_*. In the reverse bias state, the depletion region of the PN junction can significantly alleviate the electric field crowding and increase the *V_BD_*. The device with a beveled PN junction effectively combines the advantages of SBD and PND devices, and realizes the synergistic optimization strategy of large-sized wafers and high doping efficiency. In 2024, Li et al. used the chemical reflow method to form a hemispherical photoresist as a mask, and then achieved a beveled MESA with a certain curvature after dry etching (Figure 7c) [37]. The *V_BD_* increased from 188 to 229 V after the preparation of the beveled MESA, indicating that the electric field distribution became more uniform. Furthermore, depositing an HfO_2_ dielectric layer as a field plate structure can further reduce the electric field crowding and significantly increase the *V_BD_* from 291 to 380 V. The calculation shows that the maximum breakdown electric field inside the surface diode reaches 8.9 MV/cm.

The trench MOS barrier Schottky (TMBS) diode structure can be achieved using only a single doping type of semiconductor, and is often used in power devices such as Si, silicon carbide, and gallium nitride to alleviate the doping symmetry problem (Figure 7d). The forward conduction of TMBS is similar to that of the Schottky contact, which can achieve low *V_on_*. When reverse biased, the charge–coupling effect of the metal-insulator-semiconductor (MIS) structure can form a lateral depletion region in the semiconductor below the Schottky contact to achieve pinch off. For reverse-biased MIS structures, the thermal energy inside the device excites carriers and causes an inversion layer. The inversion layer will cause the entire reverse bias to fall on the insulation layer in the MIS structure, resulting in premature breakdown of the insulation layer and power devices. The only option to prevent such catastrophic failures from occurring is to quickly evacuate the minority carriers. The wide bandgap of diamond allows for a very long inversion time, effectively suppressing the formation of inversion layers in TMBS structures.

**Figure 8:**
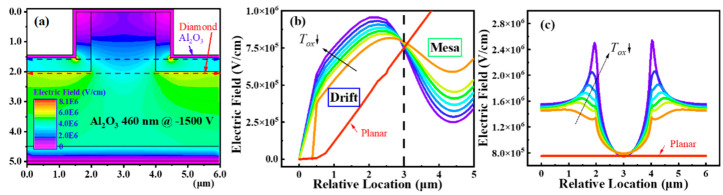
(**a**) Representative electric field distribution of the TMBS structure. (**b**) Along the vertical direction, a thinner dielectric is more conducive to transferring the electric field from the Schottky interface to the drift layer, but (**c**) in the horizontal direction, electric field peaks will occur at the corners of the MESA. Reprinted with permission from ref. [36].

Lin et al. systematically simulated the diamond TMBS structure compared to the planar type (Figure 8a). It was found that the introduction of the MIS structure can significantly reduce the electric field strength near the electrode, and the electric field peak shifts from near the Schottky contact interface to deeper drift layers [38]. MIS structures with thinner dielectric layers exhibit stronger charge coupling effects, effectively adjusting the electric field in MESA to the drift layer (Figure 8b). But it will cause the horizontal electric field in MESA to crowding at the corners of the dielectric layer (Figure 8c). A smaller MESA width is also conducive to the complete depletion of MESA by charge coupling effect, thereby significantly alleviating the electric field near the corner of the MESA. However, a too-small MESA width results in lower forward current (increased *R_on_*) in the device. Therefore, moderate dielectric layer thickness, MESA width, and MESA depth can achieve a compromise balance between electric field and current distribution. Wang et al. prepared TMBS with MESA width and depth of 2 and 200 μm using an alumina dielectric layer [39]. The forward *R_on_* is 5.6 mΩ cm^2^, and the reverse *V_BD_* of the device has been increased from 172 V to 265 V.

TMBS utilizes a dielectric layer to form a depletion region and suppress leakage current during the off-state. Therefore, the dielectric layer not only needs to have a good band alignment with diamond but also requires a matching dielectric constant and critical electric field strength. These requirements somewhat limit the choice of dielectric layer types. Secondly, diamond materials do not have an intrinsic oxide layer like SiO_2_ in silicon devices. Therefore, the existing dielectric layer is usually hetero-epitaxially grown on the surface of diamond, and factors such as lattice mismatch can lead to a large number of interface states. During device operation, interface defects can trap or release carriers, resulting in degradation phenomena such as hysteresis and a shift in electrical performance. In addition, crystalline defects inherent in the dielectric layer can lead to time-dependent dielectric breakdown under stress, resulting in device failure. How to select appropriate materials and growth methods to improve the interface state and crystallization quality of the dielectric layer is the key point in this scheme.

#### 2.3.4. The Schottky PN Diode Structure

**Figure 9:**
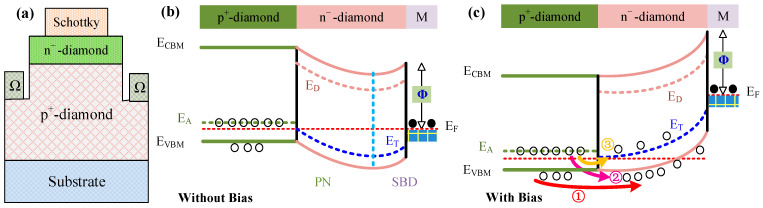
(**a**) Schematic diagram of the structure of a diamond SPND. (**b**) The band diagram under the thermal equilibrium state. (**c**) Presenting different transport mechanisms for different voltage ranges.

Although the n-type diamond has a lower carrier concentration, it can still be used in power SBD through novel device structure design. Diamond Schottky PN diode (SPND) is a device structure that utilizes n-type-doped diamond to achieve unipolar operation (Figure 9a) [40,41]. The band diagram of diamond SPND under thermal equilibrium state is shown in Figure 9b. Both p^+^/n^−^ and metal/n^−^ junctions generate depletion regions, forming a completely depleted n-type diamond drift layer. Based on the temperature-dependent current–voltage characteristics, the conduction mechanism under forward bias can be analyzed (Figure 9b) [42]. Under a small forward bias, the PN junction is non-conductive, and the trap energy level (ET) acting as a hole trap is located below the Fermi level, which does not participate in the trapping and de-trapping process. Therefore, the holes in the valence band (partially activated by boron dopants) can be driven into n-type diamond, leading to dominant Ohmic transport (process ① in Figure 9c). As the forward bias voltage increases, the PN junction gradually conducts and the diffusion current increases significantly (process ② as shown in Figure 8c). In this case, the holes are mainly provided by the thermal activation of the acceptor level formed by boron dopants. However, the increase in the energy band under forward bias also upshifts the ET. Further increase in forward bias will cause the ET to move above the Fermi level and exhibit a relatively small energy difference. Then, the holes will be transferred to the Schottky contact through ET and form charge-limited space-charge-limited current conduction (process ③ in Figure 9c). From the operation modes of the device, we can see that only holes participate in its conduction and turn off, exhibiting high current density, low specific *R_on_*, and high frequency. At the same time, the electrical characteristics of the device exhibit significant temperature dependence and can be used to prepare temperature sensors. Similarly, Liu et al. deposited a 180 nm unintentionally doped Ga_2_O_3_ film on lightly boron-doped p-type diamond by atomic layer deposition, and then deposited a 300 nm tungsten electrode to form a SPND [43]. The device exhibits good electrical characteristics in the temperature range of 298 to 523 K and can be used for in situ temperature measurements. The diode current (*I_D_*) of the SPND under a forward voltage (*V_F_*) is expressed as the following equation: [40]

(2) $$I_{D}=I_{s}\left(e^{\frac{qV_{F}}{2kT}}-1\right)=\frac{qAW}{\tau_{e}}\sqrt{N_{C}N_{V}}e^{\frac{-E_{g}}{2kT}}\left(e^{\frac{qV_{F}}{2kT}}-1\right)$$

where *A*, *W*, *N_C_, N_V_*, *τ_e_*, and *E_g_* are the device area, depletion width, effective density of conduction band states, effective density of valence band states, effective carrier life-time, and bandgap, respectively. Then, the *V_F_* decreases linearly with increasing temperature at a specific current level, with the theoretical sensitivity expressed as follows:

(3) $$\frac{dV_{F}}{dT}=\frac{2k}{q}ln\left(\frac{I_{D}\tau_{e}}{qAW}\right)-\frac{k}{q}ln\left(N_{C}N_{V}\right)=\frac{2k}{q}ln\left(\frac{I_{D}\tau_{e}}{qAW}\right)-7.54\;mV/K$$

Therefore, the sensitivity of the SPND diode sensor is proportional to the logarithm of current density.

#### 2.3.5. Junction Terminal Extension

Junction Terminal Extension (JTE) is a common termination structure in semiconductor devices such as silicon, which introduces a region with opposite doping type of the drift layer to form a PN junction next to the Schottky electrode [44]. When a certain forward bias is applied to the device, the Schottky contact with a relatively lower barrier turns on first. When a large reverse bias is applied to the SBD, the depletion region of the PN junction laterally expands to suppress the electric field crowding around the Schottky electrode and transfer it to the drift layer, thereby increasing the reverse *V_BD_* of the device. Meanwhile, due to the built-in electric field of the PN junction being higher than the Schottky junction, devices with JTE have lower leakage current during reverse bias compared to SBD. However, JTE terminals have not yet been applied in diamond, mainly due to the difficulty in achieving efficient n-type doping.

**Figure 10:**
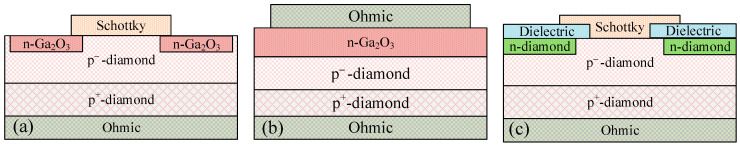
Schematic diagram of the JTE structure of vertical diamond SBDs: (**a**) the planar JTE with Ga_2_O_3_/diamond PN junction, (**b**) the vertical Ga_2_O_3_/diamond PND, and (**c**) the FP-assisted JTE structure.

An alternative solution is to form a heterojunction by searching easily n-type doped gallium oxide (Ga_2_O_3_), gallium nitride, and zinc oxide in wide bandgap semiconductor systems (Figure 10a). Ga_2_O_3_ can better match diamond compared to other n-type materials due to its large bandgap (4.8 eV) and high critical electric field (8 MV/cm). In 2022, Lin et al. simulated an SBD with a Ga_2_O_3_ JTE structure. The *V_BD_* of the device is significantly affected by the dimension and doping concentration of the Ga_2_O_3_ JTE [45,46]. Then, Wang et al. obtained GaO_x_/diamond PN heterojunction using radio frequency magnetron sputtering. The GaO_x_ film deposited on single-crystal boron-doped diamond has a roughness of 0.71 nm and a bandgap of approximately 4.85 eV. XPS spectroscopy confirmed that the GaO_x_/diamond PN heterojunction interface exhibits a type II band configuration, with valence and conduction band offsets of 1.28 eV and 1.93 eV, respectively [47]. Zhang grew 1% Sn-doped ε-Ga_2_O_3_ thin films on p-type boron-doped diamond substrate by pulsed laser deposition [48]. The Sn dopant promoted the crystallization of ε-Ga_2_O_3_ as well as achieved n-type doping. Diamond and ε-Ga_2_O_3_ exhibit excellent heterojunction PN diode characteristics (Figure 10b), with a *V_on_* of approximately 2.5 V, a rectification ratio exceeding 10^8^, and a *V_BD_* of over 3000 V. Heterojunction JTE primarily aims to serve as an alternative solution before the realization of n-type doped diamond. Typically, the two materials forming the heterojunction exhibit significant differences in crystal parameters (such as symmetry and lattice constants) and thermal expansion coefficients. This leads to stress and defects at the growth interface due to lattice constant and thermal expansion coefficient mismatches. Consequently, the primary challenge faced by heterojunction JTE is the trapping and de-trapping of carriers during device operation due to the interface defects, resulting in degradation phenomena such as hysteresis and shift in electrical performance. How to select suitable materials and growth methods in conjunction with electrical measurements to improve the interface state and long-term reliability is a key priority for this approach.

The JTE structure is more effective than FP in suppressing electric field crowding around the electrode edge, but it also increases the *R_on_* due to the reduction in conductive area. Li et al. simulated a vertical diamond SBD with FP-assisted JTE structure (FP-JTE) to achieve a balance between *R_o_*_n_ and *V_B_*_D_ (Figure 10c) [49]. The simulation results indicate that the length of JTE should be shorter than FP to enhance current conduction, but it needs to cover the edges of Schottky electrodes to suppress electric field crowding. Meanwhile, the FP-JTE SBD with JTE gradually extending from the sidewall to the center is more conducive to combining the advantages of both FP and JTE structures.

#### 2.3.6. The Junction Barrier Schottky Diode

**Figure 11:**
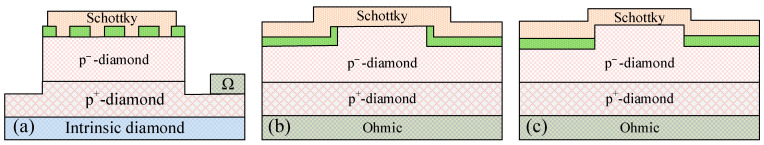
Schematic diagram of diamond JBS device structure: (**a**) planar JBS device, (**b**) trench JBS device, and (**c**) trench JBS device with sidewall reinforcement structure. The green areas represent the n-type diamond.

The junction barrier Schottky diode (JBS) selectively forms a series of n-type-doped regions on the surface of p-type-doped diamond (Figure 11), with Schottky contacts formed in the interval of the n-type-doped regions [50]. By adjusting the spacing distance between n-doped regions, Schottky contacts can be ensured to conduct at relatively low forward biases. When the device is in the off-state, the lateral depletion region provided by the PN junction can pinch off the Schottky contact and transfer the electric field peak at the interface to the drift layer. Furthermore, when the Schottky metal forms an ohmic contact with the p-type region, a Merged PN/Schottky diode (MPS) structure can be obtained [51]. When the forward voltage reaches a certain level, the PN junction will achieve secondary conduction and enhance the surge current capability [52]. Therefore, the JBS/MPS structure can combine the good forward conduction characteristics of SBD (low *V_on_*) and the reverse turn-off characteristics of PND (low leakage current and high *V_BD_*), making it a promising technical solution.

Kubovic et al. grew a thin nitrogen-doped cap layer with a thickness of approximately 10 nm on top of a low boron-doped p-type layer (Figure 11a) [53]. Then, the nitrogen-doped layer is dry etched using O_2_/Ar plasma to expose the Schottky electrode window and deposit metal. For a diode with a diameter of 100 μm, a high rectification ratio of 10^9^ is achieved at a voltage of ±8 V, and the breakdown electric field is about 2.5 MV/cm. However, the PN junction mainly generates depletion regions in the longitudinal direction and has a weak shielding effect on the Schottky contact regions. Commonly, a trench structure is formed by etching to deposit an n-type layer, and the depletion region of the PN junction in the transverse direction can significantly improve the device’s off-state performance (Figure 11b) [54]. On the other hand, suppressing the forward conduction area will increase the *R_on_* to a certain extent. In order to better balance the forward conduction and reverse off-state performance, Zhu et al. designed a trench diamond junction barrier Schottky (JBS) diode with a sidewall reinforcement structure (Figure 11c) [55]. Compared with traditional trench JBS, the Schottky contacts on the sidewalls weaken the junction field-effect transistor effect between the trenches. A slight increase in Schottky contact area can increase current density and maintain a relatively high *V_BD_*. In addition, the effects of different structural parameters (such as n-type doping concentration, MESA width, and n-type diamond thickness) on the electrical performance were comprehensively discussed. The JBS structures still face significant challenges as they rely on the improvement in n-type doping technology to achieve better device performance.

#### 2.3.7. SBD Fabricated on Non-(100) Diamond

**Figure 12:**
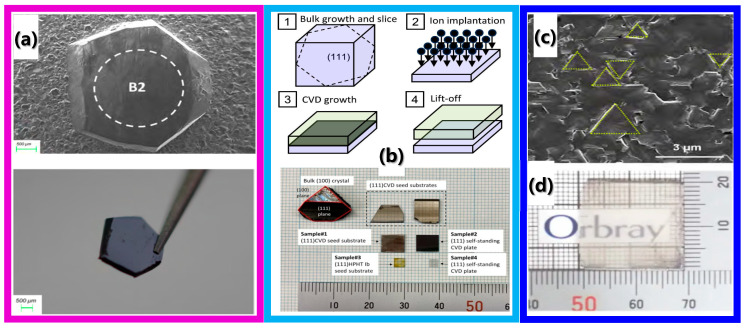
(**a**) The growth and boron-doping of the (113) diamond, (**b**) the fabrication and growth of (111) diamond, and (**c**,**d**) are the heteroepitaxy growth of (111) diamond. Reprinted with permission from refs. [61,62,63,64].

Efficient n-type diamond epitaxial layers with precisely controllable doping concentration play a crucial role in the termination structure of power diodes, and the crystal faces of diamond have a significant impact on the doping efficiency and electrical properties of the n-type doping. Numerous experimental results indicate that the doping concentration and efficiency of (113) and (111) crystal faces are 1–2 orders of magnitude higher than those of (100) crystal face [56,57]. Li et al. systematically compared the effects of diamond crystal faces and their surface termination types on phosphorus doping based on first-principles calculations [58]. The computational results clearly show that the formation energies of phosphorus doping on different crystal faces satisfy (113) < (111) < (110) < (100). This is because the (113) crystal face can be decomposed into atomic terraces of the (100) crystal face and raisers of the (111) crystal face. The terraces atoms exhibit dual symmetry and carry two dangling bonds, resulting in discrepancies in interatomic distances in different directions on the (113) crystal plane, which facilitates the incorporation of P atoms into the lattice with lower formation energy and high efficiency [59,60]. In addition, the formation of hydrogen or oxygen terminations on the surface slightly increases the formation energy of phosphorus doping and reduces doping efficiency. In 2005, C. Tavaresa et al. fabricated SBD using phosphorus-doped n-type (111) diamond, and the devices exhibited excellent rectification characteristics from 293 K to 773 K [61]. The ideal factor and rectification ratio of the devices at 573 K were 1.0 and 10^6^ (@10 V), respectively. In 2022, P. Hazdra et al. fabricated the first quasi-vertical SBD based on boron-doped p-type (113) diamond [62]. It was demonstrated that Mo could form good and stable Schottky and ohmic contacts on (113)-oriented boron-doped diamond at a wide temperature range. The forward current density, reverse current density, ideality factor, and barrier height of the device at 130 °C were 1000 A/cm^2^, 10^−8^ A/cm^2^, 1.23, and 1.71 eV, respectively, comparable to SBDs fabricated on (100) diamond. However, the research progress of SBDs was limited by the size of non-(100) SCD wafers. In 2024, J. Achard reduced the relative growth rate of the (113) crystal plane by adjusting growth parameters, and the (113) diamond was expanded from a 2 mm × 2 mm to a 3.7 mm × 3.7 mm after 260 h of growth [63] (Figure 12a). In the same year, Shimaoka et al. grew a 10 × 10 × 6 mm^3^ (100) diamond and then cut out a 7 × 6 mm^2^ (111) plane seed crystal, verifying the feasibility of replication cloning on this substrate (Figure 12b) [64]. In the future, larger-sized (111) crystal plane single crystals could be achieved through a mosaic method and heteroepitaxy technologies similar to those for (100) diamond (Figure 12c,d) [65,66].

Finally, we summarized the electrical characteristics of some typical edge termination structures for comparison (Table 1). In the literature reports, authors typically prepare diamond SBDs with and without termination structures roduction of termination structures. Of course, the existing literature reports usually only introduce one type of termination structure. Due to inconsistencies in the substrates, epitusing the same substrate, drift layer thickness, and device technology, and compare the electrical performance of SBDs with and without termination structures under the same test conditions. Therefore, we can conclude that the improvement in device performance is mainly attributed to the intaxial materials, and process parameters used in each report, it is not yet possible to conduct a lateral comparison of the effects of different termination structures. From the perspective of practical process integration, the deposition of dielectric, etching of MESA, and implantation processes can utilize the same equipment, gases, and industrial platforms as traditional gallium nitride or silicon carbide. However, due to the intrinsic material characteristics of diamond (such as bandgap width, hardness, and atomic density), the selection of deposition dielectric layers is relatively limited, the etching rate is relatively lower, and the implantation damage is relatively greater. Considering various factors, the difficulty of process implementation increases in the following order: field plate structure, metal-based guard ring, etching mesa, high-resistance guard ring, and junction termination structure (such as JTE and JBS). However, junction termination structures can achieve superior device performance after achieving breakthroughs in n-type doping.

## 3. Conclusions and Prospective

With the rapid development of the demand for carbon neutrality in society, the industrialization demand for diamond-based power devices has become increasingly urgent. In recent years, significant breakthroughs have been made in large-sized single-crystal wafers (approaching 4 inches) through mosaic growth and heteroepitaxy technologies, and their crystal quality has been continuously improved by suppressing the dislocation density with the assistance of various lateral epitaxial growth schemes. Based on this, researchers have designed and fabricated various prototype diamond power diode devices, which have exhibited excellent performance. However, the existing device performance has not fully utilized the superiority of diamond materials, and further collaborative optimization is still needed in several aspects, such as material epitaxial growth, efficient doping technology, and advanced terminal structures. On the one hand, the combination of different termination technologies and their fabrication processes needs to be optimized to further improve device performance. On the other hand, key breakthroughs are needed in n-type diamond dopants and their epitaxial growth technology, leveraging the advantages of PN junctions to further enhance the efficiency of termination structures. With the continuous improvement in the quality of large-sized diamond wafer and the progress in device design and fabrication technology, diamond-based power devices will also demonstrate even greater potential.

## Figures and Tables

**Table 1:** Electrical characteristics of some typical edge termination structures for comparison.

Termination Type	Drift Layer (nm)	Doping Concentration (cm^−3^)	SBH (eV)	R_on_ (mΩcm^2^)	V_BD_ (V)	Ref.
Field plate	500	2.69 × 10^15^	1.65	1.31	185	[23]
Metal guard ring	530	2.83 × 10^15^	1.42	/	109	[28]
Dual barrier	200	/	2.39	/	66.4	[29]
F ion implantation	350	0.91 × 10^15^	1.14	50	117	[30]
B ion implantation	310	1.0 × 10^15^	/	1.2	126	[31]
Nitrogen-doped layer	500	7.34 × 10^14^	1.28	3.16	112	[32]
Beveled MESA	1000	8.77 × 10^15^	1.52	3.0	380	[35]
TMBS	1000	3.45 × 10^15^	1.21	5.6	265	[37]

## Data Availability

Data are contained within the article.
